# Supplementary material for: Opportunities to sustain a multi-country quality of care network: Lessons on the actions of four countries Bangladesh, Ethiopia, Malawi, and Uganda
Source: PLOS Glob Public Health. 2023 Sep 12;3(9):e0001672. doi: 10.1371/journal.pgph.0001672 (PMC10497147; doi:10.1371/journal.pgph.0001672)
Supplement: Attachment — Submitted filename: PGPH_response to reviewers_10_04_23.docx [file pgph.0001672.s004.docx]

Rebuttal letter

Vanessa Carels
Staff Editor
PLOS Global Public Health

Submission date: April 10, 2023

Dear Dr. Carels,

Thank you for inviting us to address reviewer’s comments and submit a revised manuscript entitled: “Opportunities to sustain a multi-country quality of care network: lessons on the actions of four countries Bangladesh, Ethiopia, Malawi, and Uganda”
We appreciate the time and effort you and the reviewer have dedicated to providing insightful feedback. Thus, it is with great pleasure that we resubmit our article for further consideration. We have incorporated the comments and indicated the revisions we made in the manuscript. We also attached the manuscript with track change as well as the cleaned version.

The following is point by point response to the comments and questions you sent us on March 09, 2023.

Journal Requirements:

1. Please provide additional details regarding participant consent. In the ethics statement, please ensure that you have specified what type you obtained (for instance, written or verbal, and if verbal, how it was documented and witnessed). If your study included minors, state whether you obtained consent from parents or guardians. If the need for consent was waived by the ethics committee, please include this information."

**Response:** We have now addressed this issue in the Ethics session of the manuscript (Line number 208 & 210).

2. Please ensure that Funding Information and Financial Disclosure Statement are matched.

**Response:** yes, the funding information and financial disclosure statement are the same.

**Edits from our side:** we did some edits specifically on the naming of our sources of excerpts. In the earlier version we used short names now clearly wrote full names and replaced organization names by ‘partner or government’. We have indicated those changes with track change.

Reviewer #1:

1. Why were there variations in the data collection dates and the number of times data was collected for the different countries?

**Response:** Thank you for pointing this out, the study in each country received ethical approval from local institution review board at different times which created different starting dates for each country. In addition, the data collection dates and the number of times data was collected was further impacted by local COVID 19 restrictions.

2. there should be an explanation. What is the rationale behind the number of participants interviewed or the number of transcripts analyzed in the member countries.

**Response:** The main goal of participant selection was to identify key informants with the depth of information needed to answer our research questions. The final number of participants interviewed was based on the saturation level of information we were getting from the participants. We have now included a sentence describing this in the method section, line number 155 and 173-175.

3. The 1st sustainability action states "At the global level, between countries, respondents recalled the importance of holding repeat, joint international meetings with global partners" but according to table 2 of the methods supplement, global meeting minutes were not reviewed, is this because they don't exist or is there another specific reason? In Uganda also no minutes were reviewed, why?

**Response:** The QCN evaluation group had access to multiple potential sources of data and care was taken to identify the best sources to answer specific research questions. As such, for the purpose of this paper, it was decided that we use the wealth of information that we collected through the qualitative interviews and nonparticipant observations.

4. There were no observations made at local/district level meetings, would it not have been critical to this study to undertake those observations at the implementation level?

**Response:** It is true that such observations could have been a beneficial additional data collection point and we have addressed this in the limitation section line number (601-05).

5. Regarding sustainability action 3,
   It says all four countries employed various mechanisms but then only discusses one i.e small grants for health facilities what are the other types of motivating factors used and were such incentives applied in all countries, is there a quote to support that? this makes one skeptical about the all green scoring in table four for this particular theme, wouldn't you say?

**Response:** As described in that section, giving small grants as an incentive was one of the methods widely employed in three countries. The other method of motivation was the training and skill transfer opportunity that was described in line 358-59. We have now added an excerpt to support our assessment (line 360-67).

6. There were missed opportunities to ask WHY and go deeper into understanding the root causes of such findings.

**Response:** Despite our efforts to reach saturation, we agree there might be places where we could have gone deeper: this is almost always possible with qualitative methods. Nonetheless, we believe that the rich data we gathered revealed important insights. This is a multi-country study, that involved several study sites within each country, and there is a limit to how much detail can be included. However, we did show the commonalities as well as differences pertaining to the sustainability actions across countries in our analysis.

7. How do the findings inform future program design and possibly evaluation, for instance seeing that Learning is an essential pillar of the LALA framework but this study indicated that some countries were doing good in one category and others in other categories, why did they fail to learn from one another?

**Response:** We are actively engaged in discussions with global and national actors to make sure that these findings have the opportunity to inform decisions. Recently there was a global meeting in Ghana (March 14-16), and the results were presented for the global audience and the research team were engaged in a fruitful dialogue during the event. Regarding the learning between countries and within, a deeper analysis was made by the research team and submitted for publication (paper two and three).

8. With the exception of Ethiopia, all countries had at least three rounds of interviews, were there any changes in the responded across time, how is that reflected in this study?

**Response:** We didn’t fully understand this question; if it is about changes to the respondents over time, at each round we went to the same facilities and engaged the same staff but we also interviewed new people where staff changes meant that new key informants could be identified. Similarly, at the national level, we identified the main QCN actors and followed-up with them in subsequent rounds, as well as any new important national actors that had joined the network in the meantime. In our analysis, we used data from all rounds, including in Ethiopia where we had two iterative rounds of data collection and as a result were able to see how things changed over time.

Again, thank you for the detailed and valuable comment. I hope this revision satisfactorily address all the concerns you and the reviewer have raised.

Sincerely,

Seblewengel Lemma

Corresponding Author

London School of Hygiene and Tropical Medicine

[Seblewengel.abreham@lshtm.ac.uk](mailto:Seblewengel.abreham@lshtm.ac.uk)
