# Supplementary material for: Opportunities to sustain a multi-country quality of care network: Lessons on the actions of four countries Bangladesh, Ethiopia, Malawi, and Uganda
Source: PLOS Glob Public Health. 2023 Sep 12;3(9):e0001672. doi: 10.1371/journal.pgph.0001672 (PMC10497147; doi:10.1371/journal.pgph.0001672)
Supplement: Attachment — Submitted filename: PGPH_response to reviwers_04_08_23.docx [file pgph.0001672.s006.docx]

Rebuttal letter

Dario Ummarino
Staff Editor
PLOS Global Public Health

Submission date: August 04, 2023

Dear Dr. Ummarino,

Thank you for inviting us again to address reviewer’s comments and submit a revised manuscript entitled: “Opportunities to sustain a multi-country quality of care network: lessons on the actions of four countries Bangladesh, Ethiopia, Malawi, and Uganda”
We appreciate the time and effort you and the reviewer have dedicated to providing insightful feedback. Thus, it is with great pleasure that we resubmit our article for further consideration. We have incorporated the comments and indicated the revisions we made in the manuscript. We also attached the manuscript with track change as well as the cleaned version.

The following is point by point response to the comments and questions you sent us on July 13, 2023.

Journal Requirements:

1. Please provide additional details regarding participant consent. In the ethics statement, please ensure that you have specified what type you obtained (for instance, written or verbal, and if verbal, how it was documented and witnessed). If your study included minors, state whether you obtained consent from parents or guardians. If the need for consent was waived by the ethics committee, please include this information.

**Response:** this information has already been provided in the ethics session, in the method. We have stated that we obtained a written consent. Our study didn’t include minors, so we didn’t add the statement about obtaining consent from parents or guardians.

2. Please review your reference list to ensure that it is complete and correct. If you have cited papers that have been retracted, please include the rationale for doing so in the manuscript text, or remove these references and replace them with relevant current references. Any changes to the reference list should be mentioned in the rebuttal letter that accompanies your revised manuscript. If you need to cite a retracted article, indicate the article’s retracted status in the References list and also include a citation and full reference for the retraction notice.

**Response:** We have already reviewed our reference list, and we didn’t include retracted reference. We used Zotero reference manager to manage reference, and it has a feature that detect retracted papers when used.

Reviewer #2: Dear Authors,
I would like to congratulate you for an interesting and well-written manuscript.
I have very minor comments or edits I have suggested which hope you will consider for the final version of your manuscript.
I have no additional comments,
Yours sincerely.

**Response:** Thank you for the comments and the kind words. I have incorporated the comments suggested in the document as follow:

1. Line 97-99: I would suggest same aim presented in the abstract: " This paper investigated the experience of actions taken to sustain QCN in four Network countries (Bangladesh, Ethiopia, Malawi, and Uganda) and reports on lessons learned."

**Response:** thank you for capturing this, and we have addressed it as you suggested (line number 97-98)

2. On the design, perhaps need some introductory text, such as the type of study done and why?

**Response:** We provided details on the methodology in the supplement. We also provided information regarding this in line number 100-102. In this manuscript, we added a line that mentioned the overall design used for this research work (Line number: 150)

3. Line number 424, partners were providing funds? or supporting in different ways? please review!

**Response:** What we mentioned on this session was that the QI work was dependent on partner support. Here we mentioned budget to mean the monitory support. So we changed the ‘budget’ to ‘support’ (line number: 428).

Again, thank you for the detailed and valuable comment. I hope this revision satisfactorily address all the concerns you and the reviewer have raised.

Sincerely,

Seblewengel Lemma

Corresponding Author

London School of Hygiene and Tropical Medicine

[Seblewengel.abreham@lshtm.ac.uk](mailto:Seblewengel.abreham@lshtm.ac.uk)
